# Prescription medication use of United States military service members by therapeutic classification

**DOI:** 10.3389/fphar.2022.972031

**Published:** 2022-09-27

**Authors:** Joseph J. Knapik, Daniel W. Trone, Ryan A. Steelman, Emily K. Farina, Harris R. Lieberman

**Affiliations:** ^1^ Military Nutrition Division, United States Army Research Institute of Environmental Medicine, Natick, MA, United States; ^2^ Naval Health Research Center, San Diego, CA, United States; ^3^ United States Army Public Health Center, Aberdeen Proving Ground, MD, United States

**Keywords:** central nervous system agents, non-steroidal anti-inflammatory agents, antiinfective agents, analgesics and antipyretics, centrally acting skeletal muscle relaxants, antidepressants

## Abstract

**Background:** This cross-sectional study investigated the prevalence of, and factors associated with, filled prescription medications (FPMs) among United States (US) service members (SMs).

**Methods:** A stratified random sample of active duty SMs from the Air Force, Army, Marine Corps, and Navy was obtained from military workforce records. Participants (*n* = 26,680) completed a questionnaire on demographics, physical characteristics, and lifestyle factors and approved access to their FPM for the previous 6 months. FPMs were obtained from the military Pharmacy Data Transaction Service that included all prescription medications dispensed at military medical treatment facilities, abroad, at retail pharmacies in the US, and/or through mail-order programs.

**Results:** About two-thirds (65%) of SMs had ≥1 FPM in the 6 months surveillance period. Central nervous system (CNS) agents had the highest prevalence (41%), followed by anti-infective agents (20%), eye/ear/nose/throat preparations (20%), gastrointestinal drugs (18%), autonomic drugs (17%), skin and mucous membrane agents (13%), antihistamine drugs (12%), respiratory tract agents (12%) and cardiovascular drugs (9%). Among CNS agents, overall prevalence of dispensed non-steroidal anti-inflammatory drug (NSAIDs) was 30%. The odds of any FPM was independently associated with female gender, older age, higher body mass index, former tobacco use (smoking and smokeless tobacco), lower alcohol consumption, and was highest among Army, lowest among Marine Corps personnel.

**Conclusion:** In this sample of SMs, dispensing of prescription medication was high, especially NSAIDs, but dispensing of cardiovascular drugs was much lower compared to the general US population, likely because of the younger age and higher level of physical activity of SMs.

## Introduction

Prescription medications (PMs) are provided to patients for treatment of specific medical conditions and are considered important components of treatment guidelines for many types of medical disorders (APA, 2019; Grundy et al., 2019; Unger et al., 2020; ADA, 2022). Spending on PMs in the United States (US) was $348 billion in 2020, accounting for about 9% of the $4 trillion spent on all medical care in that year (Services, 2020). In the US most adults use prescription medications (Kit et al., 2012; Che et al., 2014; Kantor et al., 2015). Improvements in medical technology, advances in pharmaceutical research, modifications of clinical practice guidelines, and changes in Food and Drug Administration (FDA) regulations can change prevalence and patterns of PM use over time. Data from the National Health and Nutrition Examination Survey (NHANES) indicated that the prevalence of PM use among adults increased from 51% in 1999 to 59% in 2012 with increases in use in 11 of 18 drug classes (Kantor et al., 2015). This increase may have been at least partially fueled by an increase in direct to consumer advertising by pharmaceutical companies following Food and Drug Administration (FDA) guidance on how this advertising should be conducted (Greene and Herzberg, 2010).

Examining broad patterns of PM use in representative samples can provide useful information on the most common classes of PMs prescribed in clinical practice. An important area of interest in pharmacoepidemiology is the types and distribution of PMs offered to patients (Montastruc et al., 2019). There have been a few studies examining these PM use patterns in the United States (Kaufman et al., 2002; Kit et al., 2012; Kantor et al., 2015; NAMCS, 2018; Martin et al., 2019), but only one previous study examined PM use among United States military service members (SMs) and that study used data from 2014 (Hurt and Zhong, 2015).

The United States Department of Defense Pharmacy Data Transaction Service (PDTS) records all prescriptions dispensed to United States military SMs (PDTS, 2022). This provides an opportunity to examine comprehensive patterns of PMs filled by SMs. Combining this information with other databases allows an exploration of factors associated with these medications. The purposes of the current study were to examine the current prevalence of the therapeutic classes of PMs filled by United States military SMs and explore demographic and lifestyle factors associated with these medications. This study updates older work (Hurt and Zhong, 2015) and expands on it by examining specific characteristics related to PM.

## Materials and methods

This cross-sectional investigation was a secondary analysis of data from a study designed to investigate the effects of dietary supplements on SM health (Calvo, 2021; Knapik et al., 2021; Knapik et al., 2022). The Naval Health Research Center’s institutional review board approved the investigation and SMs electronically consented to participate by signing an informed consent document. Investigators adhered to policies and procedures for protection of human subjects as prescribed by Department of Defense Instruction 3,216.01 and the research was conducted in adherence with provisions of 32 Code of Federal Regulations Part 219.

### Sampling frame and solicitation procedures

Details of the sampling frame, solicitation of SMs, subject recruitment flow chart, sample size determination, and response bias have been previously reported (Knapik et al., 2021). Briefly, investigators requested, from the Defense Manpower Data Center, a random sample of 200,000 SMs stratified by sex (88% male and 12% female) and branch of service (Army 36%, Air Force 24%, Marines 15%, and Navy 25%). Using this sampling frame, recruitment of participants into the study involved a maximum of eight sequential contacts between investigators and SMs. The prospective participant was first sent an introductory postal letter with a $1 pre-incentive designed to increase the response rate (Church, 1993; Edwards et al., 2005). The letter also included a link to a secure website where the SM could electronically sign the consent form and complete the questionnaire. A follow-up email message after 10 days, and postcard after 3 weeks, was sent as a reminder to those who did not initially respond. If no response was received after sending the postcard, up to five additional email reminders were sent over 8 months, after which contact with the SM ended. All postal and online contacts stated that at any time the SM could decline participation and be removed from the contact list. Recruitment began in December 2018 and no further recruitment was conducted or surveys accepted after August 2019.

### Questionnaire and pharmacy data

The online survey was designed to provide information on participant demographics, physical characteristics, and lifestyle factors. Demographics included questions on gender, date of birth (for age), education level, and military service. Physical characteristics included questions on height and weight. Lifestyle characteristics included questions on the frequency and duration of aerobic and resistance training, and use of cigarettes, smokeless tobacco, and alcohol. For cigarettes and smokeless tobacco use individuals reported if they had never smoked/used, if they smoked/used but quit (and the date they quit) and if they smoked/used ≤ 3 times/week, 4–6 times/week or every day. For alcohol, SMs were asked to report on the typical amount and frequency of consumption of beer, fermented fruit drinks, wine, and liquor; examples of each were provided on the questionnaire.

Once participants were identified by completing the informed consent and questionnaire, the list of these SMs was sent to the Armed Forces Health Surveillance Branch of the Defense Health Agency (DHA). From the Defense Medical Surveillance System relational database (Rubertone and Brundage, 2002; DMSS, 2022), the DHA returned the filled prescription medications (FPMs) of the participants for the 6-month period prior to completing the questionnaire (July 2018 to March 2019, depending on when the SM completed the questionnaire). Pharmacy data was provided by the DHA from the military PDTS as 6-digit American Hospital Formulary System codes (AHFS) (AHFS, 2019) with the specific names of medications. The PDTS database included prescriptions filled at military medical treatment facilities, abroad, at retail pharmacies in the US, and/or through mail-order programs.

The AHFS is a system for classifying drugs based on physiological changes induced by the drug (therapeutic action) and mechanisms of action at the molecular level (pharmacological action). Drugs are classified by 6-digit numbers containing three levels of information. The first tier is the broadest classification and subsequent tiers are more specific. For example, the first tier number 28 identifies “central nervous system agents”; the second tier 28:08 identifies “analgesics and antipyretics”; the third tier 28:08:04 identifies “nonsteroidal anti-inflammatory agents”.

### Statistical analysis

All statistical analyses were conducted using the Statistical Package for the Social Sciences (SPSS) Version 26, 2019 (SPSS Inc, an IBM Company). Body mass index (BMI) was computed from the questionnaire responses as weight/height^2^ (kg/m^2^). Weekly duration of aerobic and resistance training (min/week) was calculated by multiplying reported weekly exercise frequency (sessions/week) by the reported duration of training (min/session). Alcohol consumption was quantified using the National Institute of Health assumption that a “standard drink” contained 17.74 ml of alcohol (NIH, 2020). Standard drinks included 12 ounces of regular beer or fermented fruit drink (5% alcohol), 8.5 ounces of higher alcohol beer (7% alcohol), 5 ounces of wine (12% alcohol), 4.25 ounces of fortified wine (15% alcohol), and 1.5 ounces of liquor (40% alcohol). Individual were considered smokers or smokeless tobacco users if they smoked or used at least 3 times per week. Cutpoints were established for aerobic exercise, resistance exercise, and alcohol use to approximate equal numbers of SMs in each category.

FPMs were classified by their first tier 2 digit codes in the AHFS Pharmacologic-Therapeutic Drug Classification (PTDC) (e.g., 08-anti-infective agent, 28—central nervous system agents). Included was a classification for “any FPM” that encompassed all PTDCs. First tier PTDCs with the highest prevalence (≥10%) were further analyzed through their third tier 6-digit codes (e.g., 08:12:06—cephalosporins, 28:08:04 - nonsteroidal anti-inflammatory agents). If no prescription was filled in a first tier PTDC it was not included as a category in tables. For prevalence calculations, an individual could be included in >1 PTDC if they had a FPM in other PTDCs, but they were only included once within a specific PTDC regardless of the number of FPMs within the 6-months surveillance period. When calculating the total number of prescriptions, all were counted including multiple refills by SMs.

FPM prevalence (percent) with 95% confidence intervals (95%CI) was calculated for each first tier PTDC for the entire sample and stratified by gender and age. Prevalence was also calculated for the entire sample third tier PTDCs where the prevalence in the first tier was ≥10%; these values are reported in the text. Prevalence calculation was (number of SMs with ≥1 FPMs in PTDC)/(total number of SMs) X 100. Univariable logistic regression examined differences in FPM prevalence (dependent variable) across various strata of the demographic and lifestyle characteristics (independent variables). A reference stratum of each characteristic was defined with an odds ratio (OR) = 1.00 and other strata of that variable were compared to the reference stratum. Where variables were ordinal (i.e., age, formal education, BMI, aerobic training duration, resistance training duration, and alcohol intake), chi-square tests for linear trend (Mantel-Haenszel statistics) were also performed. Multivariable logistic regression determined the odds of any FPM (dependent variable) adjusted for all demographic and lifestyle factors (independent variables). Since logistic regression requires complete data on all variables, only participants who completed all demographic and lifestyle items on the questionnaire were included in the multivariable analysis (*n* = 24,942; 93% of sample).

## Results

From the sample frame of 200,000 SMs, 73% (*n* = 146,365) were successfully contacted (i.e., no returned postal mail) and of these, 26,680 (18.2%) signed the informed consent and completed the questionnaire.

The 26,680 SMs filled a total of 101,013 prescriptions in the 6-months surveillance period. Table 1 displays the prevalence of FPMs by their first tier AHFS codes. Central nervous system (CNS) agents, which include narcotic and non-narcotic analgesics, had the highest prevalence (41%), followed in descending order by anti-infective agents (20%), eye/ear/nose/throat preparations (20%), gastrointestinal drugs (18%), autonomic drugs (17%), skin and mucous membrane agents (13%), antihistamine drugs (12%) and respiratory tract agents (12%). In terms of the proportion of all FPMs, CNS agents had the highest (34%), followed in descending order by eye/ear/nose/throat preparations (10%), anti-infective agents (8%), gastrointestinal drugs (8%), and skin and mucous membrane agents (7%), autonomic drugs (7%), and cardiovascular drugs (6%).

**TABLE 1:** Prescription medications filled by service members by first tier American hospital formulary service pharmacologic-therapeutic drug classifications.

American hospital formulary service codes and descriptions	Total FPM (*n*)	Proportion of all FPMs (%)	SMs with ≥1 filled prescription (*n*)	Prevalence of filled prescriptions [% (95%CI)]
4 through 96 - Any Filled Prescription Medication	101,013	100.0	17,397	65.2 (64.6–65.8)
4 - Antihistamine Drugs	4,721	4.7	3,269	12.3 (11.9–12.7)
8 - Anti-Infective Agents	8,160	8.1	5,418	20.3 (19.8–20.8)
10 - Anti-Neoplastic Agents	208	0.2	84	0.3 (0.2–0.4)
12 - Autonomic Drugs	6,704	6.6	4,467	16.7 (16.3–17.2)
20 - Blood Formation, Coagulation, and Thrombosis	296	0.3	183	0.7 (0.6–0.8)
24 - Cardiovascular Drugs	6,007	5.9	2,504	9.4 (9.1–9.8)
28 - Central Nervous System Agents	33,855	33.5	10,923	40.9 (40.3–41.5)
32 - Contraceptives	6	0.0	5	0.0 (0.0–0.0)
36 - Diagnostic Agents	142	0.1	98	0.4 (0.3–0.5)
40 - Electrolyte, Caloric, and Water Balance	834	0.8	493	1.8 (1.6–2.0)
48 - Respiratory Tract Agents	4,521	4.5	3,129	11.7 (11.3–12.1)
52 - Eye, Ear, Nose and Throat Preparations	10,408	10.3	5,378	20.2 (19.7–20.7)
56 - Gastrointestinal Drugs	7,570	7.5	4,797	18.0 (17.5–18.5)
68 - Hormones and Synthetic Substitutes	5,504	5.4	1,795	6.7 (6.4–7.0)
76 - Oxytocins	74	0.1	22	0.1 (0.1–0.1)
80 - Antitoxins, Immune Globulins, Toxoids and Vaccines	1,162	1.2	1,142	4.3 (4.1–4.5)
84 - Skin and Mucous Membrane Agents	6,896	6.8	3,489	13.1 (12.7–13.5)
86 - Smooth Muscle Relaxants	90	0.1	55	0.2 (0.2–0.3)
88 - Vitamins	1,482	1.5	998	3.7 (3.5–3.9)
92 - Miscellaneous Therapeutic Agents	1,733	1.7	1,423	5.3 (5.0–5.6)
94 - Devices	513	0.5	340	1.3 (1.1–1.4)
96 - Pharmaceutical Aids	127	0.1	80	0.3 (0.2–0.4)

Abbreviations: FPM = filled prescription medications; SM = service member; CI = confidence interval.

The CNS agent with the most prevalent third tier category was non-steroidal anti-inflammatory drugs (NSAIDs) (29.9%), followed by opiate agonist (10.1%), miscellaneous analgesics and antipyretics (9.6%), and antidepressants (7.6%). The most prevalent anti-infective agents in the third tier categories were cephalosporins (5.1%), aminoglycosides (3.6%), and quinolones (3.1%). The most prevalent eye/ear/nose/throat preparations in the third tier categories were miscellaneous anti-infectives (7.8%), miscellaneous anti-glaucoma agents (7.1%), and miscellaneous anti-inflammatory agents (4.3%). The most prevalent gastrointestinal drugs in the third tier categories were antihistamines (5.2%), protectants (5.0%), and miscellaneous gastrointestinal drugs (4.5%). The most prevalent autonomic drugs in the third tier categories were centrally acting skeletal muscle relaxants (8.0%), α- and β-adrenergic agonists (5.2%), and β-adrenergic agonists (2.5%). The most prevalent skin and mucous membrane agents in the third tier categories were scabicides and pediculicides (4.6%), corticosteroids (3.5%), and anti-bacterials (2.8%). Among antihistamine drugs, the most prevalent third tier categories were second-generation antihistamines (10.1%), ethanolamine derivatives (1.7%), and phenothiazine derivatives (1.0%). Among respiratory tract agents, the most prevalent third tier categories were antitussives (5.5%), mast cell stabilizers (5.4%), and miscellaneous respiratory agents (1.6%).

Table 2 shows the prevalence of FPMs stratified by gender within the first tier PTDCs. Women had a significantly higher odds of filling PMs in 19 of the 22 PTDC therapeutic classes (86%) including any FPM. Men had more FPMs for cardiovascular drugs. Although there were no significant gender differences for the therapeutic classes diagnostic agents, oxytocins, and antitoxins/immune globulins/toxoids/vaccines, women still had higher odds of a FPM in those PTDSs.

**TABLE 2:** Prescription medications filled by service members by first tier American hospital formulary service pharmacologic-therapeutic drug classifications - analysis stratified by gender.

American hospital formulary service codes and descriptions	Gender	SMs with ≥1 filled prescription (n)	Prevalence of filled prescriptions (% with 95%CI)	Odds ratio (women/men) with 95%CI
4 through 96 - Any Filled Prescription Medication	Men	14,321	62.2 (61.6–62.8)	3.30 (3.01–3.62)
	Women	3,076	84.4 (83.2–85.6)	
4 - Antihistamine Drugs	Men	2,520	10.9 (10.5–11.3)	2.11 (1.93–2.31)
	Women	749	20.6 (19.3–21.9)	
8 - Anti-Infective Agents	Men	4,180	18.1 (17.6–18.6)	2.32 (2.15–2.51)
	Women	1,238	34.0 (32.5–35.5)	
10 - Anti-Neoplastic Agents	Men	63	0.3 (0.2–0.4)	2.12 (1.29–3.47)
	Women	21	0.6 (0.4–0.9)	
12 - Autonomic Drugs	Men	3,640	15.8 (15.3–16.3)	1.58 (1.44–1.71)
	Women	827	22.7 (21.3–24.1)	
20 - Blood Formation, Coagulation, and Thrombosis	Men	91	0.4 (0.3–0.5)	6.54 (4.88–8.75)
	Women	92	2.5 (2.0–3.0)	
24 - Cardiovascular Drugs	Men	2,227	9.7 (9.3–10.1)	0.77 (0.68–0.88)
	Women	277	7.6 (6.7–8.5)	
28 - Central Nervous System Agents	Men	8,951	38.9 (38.3–39.5)	1.86 (1.73–1.99)
	Women	1,971	54.1 (52.5–55.7)	
32 - Contraceptives	Men	0	0	-----
	Women	5	0.1 (0.0–0.2)	
36 - Diagnostic Agents	Men	79	0.3 (0.2–0.4)	1.52 (0.92–2.52)
	Women	19	0.5 (0.3–0.7)	
40 - Electrolyte, Caloric, and Water Balance	Men	383	1.7 (1.5–1.9)	1.84 (1.49–2.28)
	Women	110	3.0 (2.5–3.6)	
48 - Respiratory Tract Agents	Men	2,470	10.7 (10.3–11.1)	1.84 (1.67–2.02)
	Women	659	18.1 (16.9–19.4)	
52 - Eye, Ear, Nose and Throat Preparations	Men	4,304	18.7 (18.2–19.2)	1.82 (1.68–1.97)
	Women	1,074	29.5 (28.0–31.0)	
56 - Gastrointestinal Drugs	Men	3,746	16.3 (15.8–17.8)	2.09 (1.93–2.26)
	Women	1,051	28.9 (27.4–30.4)	
68 - Hormones and Synthetic Substitutes	Men	570	2.5 (2.3–2.7)	19.98 (17.93–22.25)
	Women	1,225	33.6 (32.1–35.1)	
76 - Oxytocins	Men	16	0.1 (0.1–0.1)	2.37 (0.93–6.07)
	Women	6	0.2 (0.1–0.4)	
80 - Antitoxins, Immune Globulins, Toxoids, Vaccines	Men	967	4.2 (3.9–4.5)	1.15 (0.98–1.36)
	Women	175	4.8 (4.1–5.5)	
84 - Skin and Mucous Membrane Agents	Men	2,671	11.6 (11.2–12.0)	2.21 (2.02–2.41)
	Women	818	22.5 (21.1–23.9)	
86 - Smooth Muscle Relaxants	Men	31	0.1 (0.1–0.1)	4.92 (2.86–8.41)
	Women	24	0.7 (0.4–1.0)	
88 - Vitamins	Men	542	2.4 (2.2–2.6)	5.94 (5.22–6.77)
	Women	456	12.5 (11.4–13.6)	
92 - Miscellaneous Therapeutic Agents	Men	1,152	5.04.7–5.3)	1.53 (1.33–1.75)
	Women	271	7.46.6–8.3)	
94 - Devices	Men	239	1.0 (0.9–1.1)	2.72 (2.14–3.44)
	Women	101	2.8 (2.3–3.3)	
96 - Pharmaceutical Aids	Men	3	0.0 (0.0–0.0)	165.84 (52.30–525.82)
	Women	77	2.1 (1.6–2.6)	

Abbreviations: FPM = filled prescription medications; 95%CI = 95% confidence interval.

Table 3 shows the prevalence of FPMs stratified by age within the first tier PTDCs. For any FPM and for most PTDCs prevalence increased with age. Where there were exceptions (anti-infective agents, blood formation/coagulation/thrombosis agents, CNS agents, respiratory tract agents, eye/ear/nose/throat preparations, skin and mucous membrane agents, and miscellaneous therapeutic agents) there was little difference in prevalence among some of the younger age groups, but the oldest age group still had higher prevalence than the youngest except for hormones and synthetic substitutes. For oxytocins, and contraceptives, sample sizes were very small constraining inferences. The largest age difference in prevalence (youngest to oldest) was for cardiovascular drugs.

**TABLE 3:** Prescription medications filled by service members by first tier American hospital formulary service pharmacologic-therapeutic drug classifications - analysis stratified by age.

American hospital formulary service codes and descriptions	Age (yr)	SMs with ≥1 filled prescription (*n*)	Prevalence of filled prescription medications (% with 95%CI)	Odds ratio (95%CI)
4 through 96 - Any Filled Prescription Medication	18–24	2,735	58.7 (57.3–60.1)	1.00
	25–29	3,302	59.2 (57.9–60.5)	1.02 (0.94–1.10)
	30–39	7,190	65.2 (64.3–66.1)	1.32 (1.23–1.41)
	≥40	4,048	76.7 (75.6–77.8)	2.32 (2.13–2.53)
4 - Antihistamine Drugs	18–24	390	8.4 (7.6–9.2)	1.00
	25–29	537	9.6 (8.8–10.4)	1.16 (1.02–1.34)
	30–39	1,388	12.6 (12.0–13.2)	1.58 (1.40–1.77)
	≥40	928	17.6 (16.6–18.6)	2.34 (2.06–2.65)
8 - Anti-Infective Agents	18–24	922	19.8 (18.7–20.9)	1.00
	25–29	1,076	19.3 (18.3–20.3)	0.97 (0.88–1.07)
	30–39	2,251	20.4 (19.6–21.2)	1.04 (0.95–1.13)
	≥40	1,136	21.5 (20.4–22.6)	1.11 (1.01–1.23)
10 - Anti-Neoplastic Agents	18–24	4	0.1 (0.0–0.2)	1.00
	25–29	7	0.1 (0.0–0.2)	1.46 (0.42–5.00)
	30–39	32	0.3 (0.2–0.4)	3.39 (1.20–9.58)
	≥40	40	0.8 (0.6–1.0)	8.89 (3.18–24.88)
12 - Autonomic Drugs	18–24	621	13.3 (12.3–14.3)	1.00
	25–29	786	14.1 (13.2–15.0)	1.07 (0.95–1.19)
	30–39	1892	17.2 (16.5–17.9)	1.35 (1.22–1.49)
	≥40	1,148	21.8 (20.7–22.9)	1.81 (1.63–2.01)
20 - Blood Formation, Coagulation, and Thrombosis	18–24	23	0.5 (0.3–0.7)	1.00
	25–29	23	0.4 (0.2–0.6)	0.83 (0.47–1.49)
	30–39	66	0.6 (0.5–0.7)	1.21 (0.75–1.95)
	≥40	67	1.3 (1.0–1.6)	2.59 (1.61–4.17)
24 - Cardiovascular Drugs	18–24	71	1.5 (1.201.8)	1.00
	25–29	153	2.7 (2.3–3.1)	1.82 (1.37–2.42)
	30–39	889	8.1 (7.6–8.6)	5.66 (4.44–7.23)
	≥40	1,324	25.1 (23.9–26.3)	21.66 (17.00–27.60)
28 - Central Nervous System Agents	18–24	1744	37.4 (36.0–38.8)	1.00
	25–29	1974	35.4 (34.1–36.7)	0.91 (0.84–0.99)
	30–39	4,402	39.9 (39.0–40.8)	1.11 (1.04–1.19)
	≥40	2,711	51.4 (50.1–52.7)	1.77 (1.63–1.92)
32 - Contraceptives	18–24	3	0.1 (0.0–0.2)	1.00
	25–29	1	0.0 (0.0–0.0)	0.28 (0.03–2.68)
	30–39	1	0.0 (0.0–0.0)	0.14 (0.01–1.35)
	≥40	0	0.0 (0.0–0.0)	-----
36 - Diagnostic Agents	18–24	6	0.1 (0.0–0.2)	1.00
	25–29	8	0.1 (0.0–0.2)	1.11 (0.39–3.21)
	30–39	30	0.3 (0.2–0.4)	2.12 (0.88–5.09)
	≥40	50	0.9 (0.6–1.2)	7.42 (3.18–17.33)
40 - Electrolyte, Caloric, and Water Balance	18–24	28	0.6 (0.4–0.8)	1.00
	25–29	38	0.7 (0.5–0.9)	1.13 (0.70–1.85)
	30–39	170	1.5 (1.3–1.7)	2.59 (1.73–3.87)
	≥40	240	4.5 (3.9–5.1)	7.89 (5.32–11.70)
48 - Respiratory Tract Agents	18–24	483	10.4 (9.5–11.3)	1.00
	25–29	587	10.5 (9.7–11.3)	1.01 (0.90–1.16)
	30–39	1,292	11.7 (11.1–12.3)	1.15 (1.03–1.28)
	≥40	745	14.1 (13.2–15.0)	1.42 (1.26–1.61)
52 - Eye, Ear, Nose and Throat Preparations	18–24	931	20.0 (18.9–21.1)	1.00
	25–29	1,018	18.2 (17.2–19.2)	0.89 (0.81–0.99)
	30–39	2,100	19.0 (18.3–18.7)	0.94 (0.86–1.03)
	≥40	1,274	24.2 (23.0–25.4)	1.28 (1.16–1.40)
56 - Gastrointestinal Drugs	18–24	666	14.3 (13.3–15.3)	1.00
	25–29	816	14.6 (13.7–15.5)	1.02 (0.92–1.15)
	30–39	1950	17.7 (17.0–18.4)	1.28 (1.17–1.42)
	≥40	1,322	25.1 (23.9–26.3)	2.01 (1.81–2.22)
68 - Hormones and Synthetic Substitutes	18–24	352	7.6 (6.8–8.4)	1.00
	25–29	347	6.2 (5.6–6.8)	0.82 (0.70–0.95)
	30–39	655	5.9 (5.5–6.3)	0.77 (0.68–0.88)
	≥40	423	8.0 (7.3–8.7)	1.07 (0.92–1.24)
76 - Oxytocins	18–24	2	0.0 (0.0–0.0)	1.00
	25–29	7	0.1 (0.0–0.2)	2.93 (0.61–14.09)
	30–39	9	0.1 (0.0–0.2)	1.90 (0.41–8.81)
	≥40	4	0.1 (0.0–0.2)	1.77 (0.32–9.65)
80 - Antitoxins, Immune Globulins, Toxoids, Vaccines	18–24	93	2.0 (1.6–2.4)	1.00
	25–29	229	4.1 (3.6–4.6)	2.10 (1.65–2.68)
	30–39	563	5.1 (4.7–5.5)	2.64 (2.12–3.30)
	≥40	253	4.8 (4.2–5.4)	2.47 (1.95–3.16)
84 - Skin and Mucous Membrane Agents	18–24	553	11.9 (11.0–12.8)	1.00
	25–29	620	11.1 (10.3–11.9)	0.92 (0.82–1.05)
	30–39	1,369	12.4 (11.8–13.0)	1.05 (0.94–1.17)
	≥40	917	17.4 (16.4–18.4)	1.56 (1.40–1.75)
86 - Smooth Muscle Relaxants	18–24	27	0.6 (0.4–0.8)	1.00
	25–29	37	0.7 (0.5–0.9)	1.15 (0.70–1.88)
	30–39	88	0.8 (0.6–1.0)	1.38 (0.90–2.13)
	≥40	51	1.0 (0.7–1.3)	1.68 (1.05–2.68)
88 - Vitamins	18–24	117	2.5 (2.1–2.9)	1.00
	25–29	184	3.3 (2.8–3.8)	1.33 (1.05–1.68)
	30–39	405	3.7 (3.3–4.1)	1.48 (1.20–1.82)
	≥40	284	5.4 (4.8–6.0)	2.21 (1.78–2.75)
92 - Miscellaneous Therapeutic Agents	18–24	244	5.2 (4.6–5.8)	1.00
	25–29	243	4.4 (3.9–4.9)	0.82 (0.69–0.99)
	30–39	522	4.7 (4.3–5.1)	0.90 (0.77–1.05)
	≥40	398	7.5 (6.8–8.2)	1.48 (1.25–1.74)
94 - Devices	18–24	28	0.6 (0.4–0.8)	1.00
	25–29	38	0.7 (0.5–0.9)	1.13 (0.69–1.84)
	30–39	107	1.0 (0.8–1.2)	1.62 (1.07–2.49)
	≥40	150	2.8 (2.4–3.2)	4.84 (3.23–7.26)
96 - Pharmaceutical Aids	18–24	7	0.2 (0.1–0.3)	1.00
	25–29	6	0.1 (0.0–0.2)	0.72 (0.24–2.13)
	30–39	30	0.3 (0.2–0.4)	1.81 (0.80–4.13)
	≥40	33	0.6 (0.4–0.8)	4.19 (1.85–9.47)

Abbreviation: 95% CI = 95% confidence interval.

Table 3 shows the association between any FPM and demographic and lifestyle factors. Women filled more prescriptions than men in both univariable and multivariable analyses. After eliminating SMs who filled prescriptions for contraceptive (class 32) and hormones and synthetic substitutes (class 68), women still had a higher prevalence of any FPM (men = 61.2%, women = 76.3%, OR = 2.04, 95%CI = 1.85–2.25). Filling prescriptions increased with increasing age and BMI in both univariable and multivariable analyses. As formal education increased in univariable analysis so did the odds of a FPM, but this relationship was attenuated in the multivariate analysis. In the univariable and multivariable analyses, those who smoked but quit had a higher odds of FPMs compared to never smokers. In univariable analysis, smokers had a lower odds of FPMs than never smokers, but this relationship was not significant in the multivariable analysis. For smokeless tobacco, univariable analysis showed that those who never used and those who used but quit has a similar odds of FPM, but users had a lower odds than never users. However, in the multivariable analysis never users and users had similar odds of FPMs, while those who used but quit had higher odds of a FPM. The odds of filling prescription medications decreased as alcohol intake increased in both univariable and multivariable analyses. Those performing more aerobic exercise had a marginally higher odds of filling a prescription medication in univariable analysis, but this relationship was not significant in the multivariable analysis. As the duration of resistance training exercise increased, the odds of a FPM decreased in the univariate analysis, but this relationship was not significant in the multivariable analysis. When compared to Marine Corps personnel, Army and Air Force personnel had higher odds of a FPM in both univariable and multivariable analyses. Mantel-Haenszel tests indicated significant linear trends for FPMs prevalence by age, education, BMI, alcohol use, and resistance training (all *p* < 0.01), but not for aerobic exercise (*p* = 0.07).

## Discussion

The present study involving a large sample of SMs (>26,000) found that 65% of SMs had at least one FPM in a 6-month period. When FPMs were placed into AHFS PTDCs, the most often used drug classes (with prevalence percentages in parentheses) were CNS agents (41%), anti-infective agents (20%), eye/ear/nose/throat preparations (20%), gastrointestinal drugs (18%), and autonomic drugs (17%). When drugs were further sub-classified by their tier PTDCs those with the highest prevalence included NSAIDs (30%), miscellaneous analgesics and antipyretics (10%), centrally acting skeletal muscle relaxants (8%), miscellaneous anti-infectives (8%), antidepressants (8%), miscellaneous anti-glaucoma agents (7%), α- and β-adrenergic agonists (5%), antihistamines (5%), cephalosporins (5%), and protectants (5%). FPMs were independently associated with female gender, older age, higher BMI, former tobacco use (smoking and smokeless tobacco), lower alcohol consumption, and service in the Army or Air Force compared to the Marine Corps. A large majority of ambulatory visits in the military services are accounted for by injuries and diseases of the musculoskeletal system (MSMR, 2021a) at least partly accounting for the high uses of CNS agents, especially NSAIDs.

### Prevalence of FPMs

The prevalence of any FPM in the present sample (65%) was generally higher than in nationally representative civilian samples. One study (Kaufman et al., 2002) in 1998–1999 found that 50% of Americans had used a PM in the last week. Two studies (Kit et al., 2012; Farina et al., 2014) using 2004–2008 data from NHANES found that 56 and 57% of Americans had taken ≥1 PM in the last 30 days. Other NHANES studies (Kantor et al., 2015; Randhawa et al., 2017) that examined temporal trends found increases in PM prevalence from 1988 to 2012, with one study (Kantor et al., 2015) reporting an increase from 51 to 59% from 1999–2000 to 2011–2012. The difference in prevalence estimates between NHANES data and the current study may be at least partly due to demographics, methodological differences, and availability of PMs in civilian versus military health care systems. Compared to the NHANES, the current military sample was younger and predominately male. NHANES obtained PM information from a face-to-face interview and asked about PMs used in the last 30 days while in the current study the data was obtained from comprehensive pharmacy records over the last 6 months. In the military, all PMs are provided to SMs free of charge, while PMs may be more difficult for some Americans to obtain depending on their cost, copay arrangements, and insurance coverage (Pagan and Pauly, 2005; Goldstein et al., 2014).

There has only been one previous study (Hurt and Zhong, 2015) of FPMs by PTDCs in the US military health care system. That study (Hurt and Zhong, 2015) involved the entire US military population (including the Coast Guard) in 2014 and used the PDTS database. In agreement with the present study, Hurt and Zhoung (Hurt and Zhong, 2015) found that CNS agents had the highest prevalence followed by anti-infective agents and eye, ear, nose and throat preparations; CNS agents accounted for 38% of all filled prescriptions, similar to the 34% found in the current study. In Hurt and Zhoung’s (Hurt and Zhong, 2015) third tier classification of CNS agents, NSAIDs, opiate agonists, and analgesics/antipyretics were those with the highest use, also in agreement with the present study. These data indicate similarities in the prevalence of FPMs by PTDCs in 2018–2019 and 2014.

Comparisons of specific PTDCs in the present study with that of previous studies involving representative samples of the US population are complicated by the use of different categorization systems, methods of data collection, and diverse reporting periods (i.e., surveyed periods of use). In addition, military access to healthcare and PMs are totally free of charge (no copays), as mentioned earlier. Kantor et al. (Kantor et al., 2015) analyzed 2011–2012 NHANES data, which involved PM use in the last 30 days by asking respondents to show prescription containers or report medication names. Drugs were classified by National Center for Health Statistics drug categories, although several other categories were added by the authors. Kantor et al. (Kantor et al., 2015) found that cardiovascular drug prescriptions were the most prevalent in the US population with 27% using antihypertensives and 18% using antihyperlipidemic agents; analgesics were reported by 11% and antidepressants by 13% of the US population. These results are similar to another analysis (Kit et al., 2012) of 2005–2008 NHANES data that found, in order of prevalence, the most commonly reported drug classes were cardiovascular drugs (for hypertension and lipid lowering), analgesics, and antidepressives. The National Ambulatory Medical Care Survey included 30 sampled visits from physicians at office-based practices during a randomly selected 1-week period. In 2018, the drugs most often ordered or provided to patients were analgesics, antihyperlipidemic agents, antidepressants, antidiabetic agents, and vitamins (NAMCS, 2018). The Medical Expenditure Panel Survey (MEPS, 2018) involved a household interview in which individuals were asked about their current prescription medication use and these were grouped by therapeutic classes using the Cerner Multum Therapeutic categories. The five classes (in descending order) with the highest use prevalence were CNS agents, cardiovascular drugs, anti-infectives, metabolic agents, and hormones/hormone modifiers. While cardiovascular drugs were prominent in these previous surveys (Kit et al., 2012; Kantor et al., 2015; MEPS, 2018; NAMCS, 2018), only 9% of SMs filled prescriptions within this PTDC in the current study, ranking ninth among all PTDCs. On the other hand, CNS agents (especially analgesics like NSAIDs and opiate agonists), anti-infectives, and anti-depressives were more commonly reported in these surveys, largely in agreement with the current study. The lower use of cardiovascular drugs among SMs is likely associated with the younger age of SMs since cardiovascular problems are more likely to be diagnosed among older individuals (North and Sinclair, 2012).

### Demographic and lifestyle factors associated with FPMs

Data from NHANES (Kantor et al., 2015; Randhawa et al., 2017) and other nationally representative surveys (Kaufman et al., 2002; Gardiner et al., 2006) indicated that women were more likely to report PM use than men and that reporting of PM use increased with age, in agreement with the current study. In the current study, a relationship between formal educational level and FPM was noted in the univariable analysis, but that relationship was considerably attenuated in the multivariable analysis. PMs are provided by health care professionals for specific medical conditions and the higher prevalence of FPMs among women and older SMs may be at least partly accounted for by their higher medical utilization. Women are more likely than men to seek health care in both military (MSMR, 2021b; a) and civilian populations (Muller, 1986; Bertakis et al., 2000; Ladwig et al., 2000; Friberg et al., 2016) even after accounting for pregnancy-related conditions and socioeconomic characteristics (Bertakis et al., 2000; Friberg et al., 2016; MSMR, 2021b; a). As a result, women may be more likely to receive PMs for diagnosed health problems. It was noteworthy that women had a higher prevalence of FPMs in most therapeutic classes. Even after excluding individuals filling prescriptions for hormones and synthetic substitutes (which include birth control medications) women had more than twice the odds of filling any PM. The increase in FPMs with age is also likely related to the age-related increase in diagnosed medical conditions (Ladwig et al., 2000; Atella et al., 2018; NCHS, 2018) leading to more prescribed medications. While the association between education and FPMs differed in the univariable and multivariable analyses, it is known that individuals who have achieved higher educational levels are generally more health conscious and more likely to explore multiple channels of information related to their health (Kim et al., 1994; Dutta-Bergman, 2004; Iversen and Kraft, 2006; Harper and Lynch, 2007; Ouwehand et al., 2009) which could also increase their health care utilization and PM use.

The association between higher BMI levels and greater prevalence of FPMs found in the current study has also been reported in nationally (Kit et al., 2012; Randhawa et al., 2017) and regionally (Che et al., 2014) representative samples in the US. Obesity is associated with numerous health problems including hypertension, hypercholesterolemia, Type 2 diabetes, cardiovascular disease, gallbladder disease, certain cancers, and other chronic health problems (Field et al., 2001). Overweight (BMI 25.0–29.9 kg/m^2^) and obese (BMI ≥30.0 kg/m^2^) individuals utilize more health care than their normal weight (BMI 18.5–24.9 kg/m^2^) counterparts (Bertakis and Azari, 2006; McDowell et al., 2006; Shiozawa et al., 2019). In a study (Shiozawa et al., 2019) of medical encounters among Army soldiers in 2015, the average number of visits to health care providers were 11, 13, and 20 for those with BMIs of 18.5–24.9, 25.0–29.9 and ≥30.0 kg/m^2^, respectively. The International Classification of Diseases diagnostic categories with the largest differences between normal (18.5–24.9 kg/m^2^) and obese (≥30.0 kg/m^2^) soldiers were musculoskeletal system and connective tissue disorders, mental health, and endocrine/nutritional/metabolic disorders (Shiozawa et al., 2019).

It was surprising that in the multivariable analyses the prevalence of FPMs was similar among current tobacco users (smokers and smokeless tobacco users) and those who had never used. A causal relationship has been established between smoking and many types of medical problems including cardiovascular disease, certain cancers, respiratory diseases, reproductive health problems, and other health maladies (CDC, 2004). Health risks among smokeless tobacco users are less clear, likely because of different methods of preparation of smokeless tobacco and methodological problems like not accounting for quantity and duration of use, dual smoking and smokeless tobacco use, and prior smoking (Phillips and Heavner, 2009; Clarke et al., 2019; Hajat et al., 2021). Nonetheless, health problems requiring PMs might be expected for both types of tobacco users leading to increasing PM use. In contrast to current tobacco users, those who had quit smoking or using smokeless tobacco had a higher prevalence of FPMs than never smokers in the multivariable analyses. A similar (but not significant) finding was noted in a study involving a regionally representative sample of the state of Wisconsin (Che et al., 2014). Smokers cite numerous reasons for giving up smoking, but the primary reason is health concerns (McCaul et al., 2006). In this generally younger sample, many tobacco users may not have yet developed disorders requiring PMs, but those who quit smoking may have done so because of medical problems necessitating PMs.

The reduction in the prevalence of FPMs with increasing alcohol consumption may be associated with patients voluntarily reducing consumption when taking PMs. Many PMs interact with alcohol by interfering with metabolism of the drugs through mechanisms involving absorption, distribution, metabolism, or excretion, or by enhancing the influence of PMs at the effector site (Johnson and Seneviratne, 2014). There are numerous classes of PMs that interact with alcohol including CNS drugs, anti-infectives, antihistamines, and other drug classes (Weathermon and Crabb, 1999; Johnson and Seneviratne, 2014). Thus, drug information labels, physicians, pharmacists, and other health care professionals often advise patients to reduce or abstain from alcohol while using specific PMs.

The lack of association between FPMs and the duration of weekly aerobic exercise and resistance training in the multivariable analyses was surprising because higher levels of physical activity are generally associated with lower health care utilization and health care costs (Wetzler and Cruess, 1975; Rocca et al., 2015; George et al., 2017; Kang and Xiang, 2017). One nationally representative sample of United States adults aged >18 years found a lower incidence of PM use among those who spent ≥90 min/wk of moderate to vigorous physical activity when compared to those who were physically inactive (Kang and Xiang, 2017). In the military, virtually all individuals perform regular exercise to pass the frequent physical fitness tests (Department of Defense (DoD), 2004). In addition, many military occupational specialties also involve heavy physical activity (Sharp et al., 1998). There may be little difference in FPM prevalence by exercise duration because virtually all SMs are physically active compared to civilian populations and the large majority likely meet or exceed the minimal physical activity standards for maintaining health (Garber et al., 2011).

### Strengths and limitations

The current study recruited a large stratified random sample of SMs from all branches of the United States military. The pharmacy database used in this study contained virtually complete information on medications dispensed to SMs thus providing a comprehensive assessment of FPMs provided to SMs. However, filling a prescription does not imply conformity with the prescription regime and data on actual compliance was not available. The FPMs examined here were only those prescribed by medical care providers and obtained through pharmacy channels. Medications that SMs obtained over-the-counter without a prescription (e.g., a SM went to a commercial drug store for a medication and paid out-of-pocket) were not included. Nonetheless, prescriptions provided to active duty military personnel are free of charge so SMs are more likely to use the medical/pharmacy route to obtain medications than civilians making comparisons to civilian data challenging. Another limitation was that compared with the requested stratified sample (12% female, and 36% Army, 24% Air Force, 15% Marines Corps, 25% Navy) respondent demographics differed slightly (Table 4). Nonetheless, both genders and all service branches were well represented given the large sample size.

**TABLE 4:** Association between filled prescription medications and demographic and lifestyle factors.

Variable	Strata	Unadjusted (univariable)			Adjusted (multivariable)	
		Sample size (*n*)	Prevalence (%±SE)	Odds ratio (95%CI)	Sample size (*n*)	Odds ratio (95%CI)
Sex	Men Women	23,037 3,642	62.2 ± 0.3 84.4 ± 0.6	1.00 3.30 (3.01–3.62)	21,517 3,425	1.00 3.65 (3.30–4.03)
Age	18–24 years 25–29 years 30–39 years ≥40 years	4,660 5,580 11,030 5,275	58.7 ± 0.7 59.2 ± 0.7 65.2 ± 0.5 76.7 ± 0.6	1.00 1.02 (0.94–1.10) 1.32 (1.23–1.41) 2.32 (2.13–2.53)	4,325 5,252 10,385 4,980	1.00 1.00 (0.92–1.10) 1.29 (1.18–1.41) 2.34 (2.10–2.60)
Formal Education	Some HS/HS Grad Some College College Degree	3,879 11,378 11,417	58.4 ± 0.8 66.1 ± 0.4 66.6 ± 0.4	1.00 1.39 (1.29–1.50) 1.42 (1.32–1.53)	3,576 10,667 10,699	1.00 1.10 (1.01–1.20) 0.94 (0.85–1.03)
Body Mass Index	< 25.0 kg/m^2^ 25.0–29.9 kg/m^2^ ≥30.0 kg/m^2^	7,857 13,897 4,424	61.6 ± 0.6 65.0 ± 0.4 72.0 ± 0.7	1.00 1.16 (1.09–1.23) 1.60 (1.48–1.73)	7,535 13,212 4,195	1.00 1.21 (1.14–1.29) 1.60 (1.47–1.75)
Smoking	Never Smoked Smoked but Quit Smoker	16,706 4,767 4,511	65.0 ± 0.4 68.3 ± 0.7 62.7 ± 0.7	1.00 1.16 (1.08–1.24) 0.90 (0.84–0.97)	16,118 4,504 4,320	1.00 1.16 (1.07–1.25) 1.06 (0.98–1.14)
Smokeless Tobacco	Never Used Used but Quit User	20,378 2,047 756	65.5 ± 0.4 66.7 ± 0.7 61.8 ± 0.7	1.00 1.06 (0.96–1.17) 0.85 (0.79–0.92)	19,907 1,991 3,044	1.00 1.12 (1.01–1.25) 0.98 (0.90–1.06)
Alcohol Use	None 0.23–24.85 ml/wk 24.86–71.69 ml/wk >71.69 ml/wk	8,372 6,132 6,108 6,067	68.1 ± 0.5 66.6 ± 0.6 63.1 ± 0.6 62.0 ± 0.6	1.00 0.93 (0.87–1.00) 0.80 (0.75–0.86) 0.77 (0.72–0.82)	7,434 5,849 5,868 5,791	1.00 0.88 (0.81–0.95) 0.84 (0.78–0.90) 0.80 (0.74–0.87)
Aerobic Exercise	≤90 min/wk 91–180 min/wk 181–300 min/wk >300 min/wk	7,286 7,285 5,869 6,240	63.9 ± 0.6 66.1 ± 0.6 65.4 ± 0.6 65.6 ± 0.6	1.00 1.10 (1.02–1.18) 1.07 (1.00–1.15) 1.08 (1.01–1.16)	6,488 6,959 5,576 5,919	1.00 1.03 (0.95–1.11) 0.98 (0.90–1.06) 1.01 (0.93–1.10)
Resistance Exercise	< 45 min/wk 46–135 min/wk 136–300 min/wk >300 min/wk	7,776 6,257 6,581 6,066	67.2 ± 0.5 66.1 ± 0.6 63.9 ± 0.6 63.0 ± 0.6	1.00 0.95 (0.89–1.02) 0.86 (0.81–0.93) 0.83 (0.77–0.89)	6,881 5,969 6,283 5,809	1.00 0.98 (0.90–1.05) 0.95 (0.88–1.03) 0.96 (0.89–1.05)
Service Branch	Marine Corps Air Force Navy Army	3,194 9,788 5,763 7,935	56.6 ± 0.9 65.0 ± 0.5 63.4 ± 0.6 70.2 ± 0.5	1.00 1.42 (1.31–1.54) 1.33 (1.21–1.45) 1.80 (1.66–1.96)	2,964 9,233 5,364 7,381	1.00 1.18 (1.08–1.30) 0.99 (0.90–1.10) 1.43 (1.30–1.57)

abrAbbreviationsSE = standard error; 95%CI = 95%confidence interval; HS = high school.

## Conclusion

In the current study, 65% of SMs had ≥1 FPM in a 6-month period. The most often filled prescriptions were in drug classes CNS agents (41%), anti-infective agents (20%), and eye/ear/nose/throat preparations (20%). FPMs were independently associated with female gender, older age, higher BMI, former use of tobacco (smoking and smokeless tobacco), lower alcohol consumption, and service in the Army or Air Force compared to the Marine Corps. Comparisons with the previous military study (Hurt and Zhong, 2015) suggests little change since 2014 in the most commonly dispensed PTDCs, with CNS agents accounting for over one third of all FPMs, and non-steroidal anti-inflammatory agents accounted for a large proportion of those CNS agents. Comparisons with civilian studies suggest military personnel have a much lower use of cardiovascular drugs, likely because of the younger and healthier military population. This study provides basic epidemiological information on the prevalence of PTDCs dispensed to SMs and provides demographic and lifestyle factors associated with these dispensed medications.

## Data Availability

The raw data supporting the conclusion of this article will be made available by the authors, without undue reservation.

## Author disclaimer

We are employees of the United States Government. This work was prepared as part of our official duties. Title 17, U. S. C. §105 provides that copyright protection under this title is not available for any work of the United States Government. Title 17, U. S. C. §101 defines a United States Government work as work prepared by a military service member or employee of the United States Government as part of that person’s official duties. This work was supported by the Department of Defense Center Alliance for Nutrition and Dietary Supplement Research of the Defense Medical Research and Development Program, the United States Army Medical Research and Development Command (USAMRDC) under work unit no. N1335. The views expressed in this article are those of the authors and do not necessarily reflect the official policy or position of the Department of the Navy, Department of Defense, nor the United States Government. The study protocol was approved by the Naval Health Research Center Institutional Review Board in compliance with all applicable Federal regulations governing the protection of human subjects. Research data were derived from an approved Naval Health Research Center, Institutional Review Board protocol number NHRC.2016.0025.
